# Supplementary material for: Impacts of Telomeric Length, Chronic Hypoxia, Senescence, and Senescence-Associated Secretory Phenotype on the Development of Thoracic Aortic Aneurysm
Source: Int J Mol Sci. 2022 Dec 7;23(24):15498. doi: 10.3390/ijms232415498 (PMC9779024; doi:10.3390/ijms232415498)
Supplement: Supplementary file 1 [file ijms-23-15498-s001.zip › ijms-2031417-supplementary.pdf]

Supplementary Figures

A

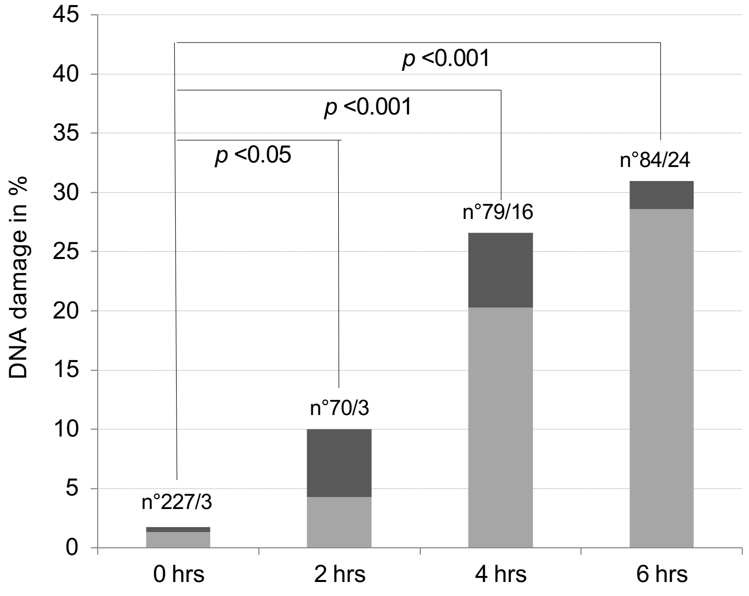

B

time

	Media	
time	γH2AX positive %	TIF positive %
0 hrs	1.32	0.44
2 hrs	4.29	5.71
4 hrs	20.25	6.33
6 hrs	28.57	2.38

C

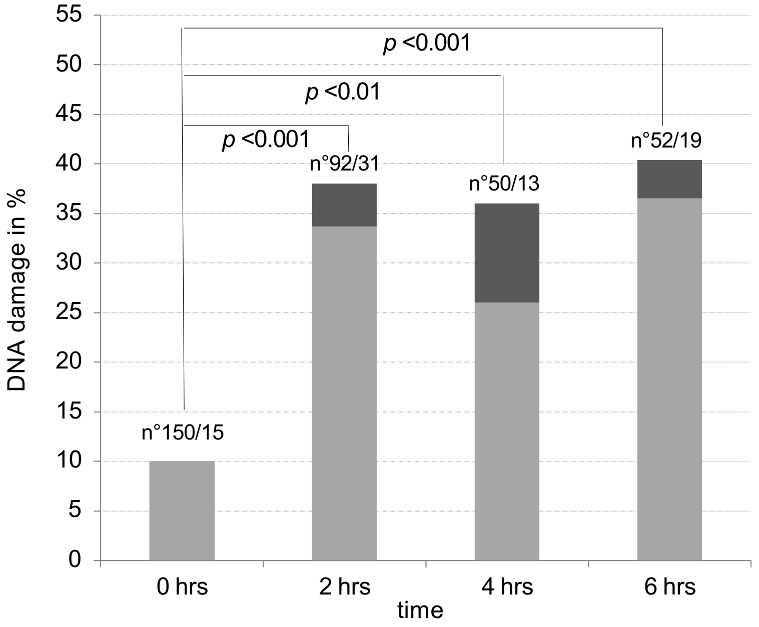

D

	Endothel/Intima	
time	γH2AX positive %	TIF positive %
0 hrs	10.00	0.00
2 hrs	33.70	4.35
4 hrs	26.00	10.00
6 hrs	36.54	3.85

Figure S1. Incorrect sample preparation leads to false-positive DNA damage signals. We analysed DNA damage response signals on different time-points by γH2AX and TIF immunohistostaining to avoid false-positive results. Tissue was immediately fixed during surgery, or after 2, 4, and 6 hours incubation in PBS. TIF signals (dark grey) were measured by co-localisation staining of TRF-1 and γH2AX signals (grey). Illustrating the positive n° of γH2AX in the n° of nuclei counted. Statistical analysis wer seperatly calculated for (A+B) medial layer, and (C+D) endothelial/intima layer. Analysis was performed by two-tailed Student *t*-test. Tables show values in %. Assays were done on healthy aortic tissue specimen (n=5).

A

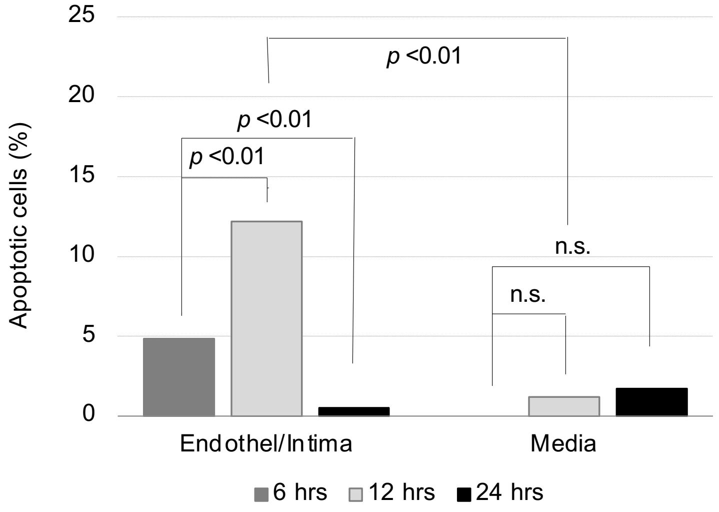

B

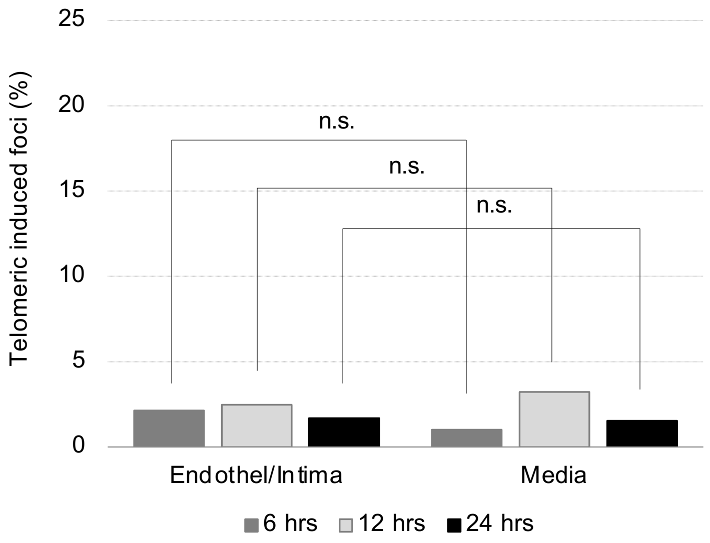

Figure S2. Hypoxic ex-vivo conditions lead to an increase of apoptotic cells, but unaltered telomeric ends. Human tissues were stored in cell culture medium for 6, 12, or 24 hours (hrs), or fixed immediately during surgery. (A) Apoptotic cells, as well as, (B) telomeric induced foci were analysed by immunofluorescence. Endothelial/intimal and medial layer are shown separately. Statistical significance are given (n=5, ANOVA). n.s., non-significant.
